# Advances in risk stratification of bladder cancer to guide personalized medicine

**DOI:** 10.12688/f1000research.14903.1

**Published:** 2018-07-25

**Authors:** Justin T. Matulay, Ashish M. Kamat

**Affiliations:** 1 Department of Urology, Division of Surgery, University of Texas MD Anderson Cancer Center, 1515 Holcombe Blvd, Suite 853, Houston, TX, 77030, USA

**Keywords:** Bladder cancer, bladder cancer genetics, personalized medicine, risk stratification, urothelial carcinoma

## Abstract

Bladder cancer is a heterogeneous disease that poses unique challenges to the treating clinician. It can be limited to a relatively indolent papillary tumor with low potential for progression beyond this stage to muscle-invasive disease prone to distant metastasis. The former is best treated as conservatively as possible, whereas the latter requires aggressive surgical intervention with adjuvant therapies in order to provide the best clinical outcomes. Risk stratification traditionally uses clinicopathologic features of the disease to provide prognostic information that assists in choosing the best therapy for each individual patient. For bladder cancer, this informs decisions regarding the type of intravesical therapy that is most appropriate for non-muscle-invasive disease or whether or not to administer neoadjuvant chemotherapy prior to radical cystectomy. More recently, tumor genetic sequencing data have been married to clinical outcomes data to add further sophistication and personalization. In the next generation of risk classification, we are likely to see the inclusion of molecular subtyping with specific treatment considerations based on a tumor’s mutational profile.

## Introduction

Bladder cancer ranks among the most common non-cutaneous malignancies in the United States (4 ^th^ for men and 11 ^th^ for women) and worldwide (6 ^th^ for men and 16 ^th^ for women) ^1– 3^. Though it is commonly referred to by the generic term “bladder cancer”, urothelial neoplasms represent a broad spectrum of disease with vastly different treatment pathways from routine bladder surveillance to intravesical therapy to radical surgery with chemotherapy. Staging is broadly divided into two major categories—non-muscle-invasive bladder cancer (NMIBC) and muscle-invasive bladder cancer (MIBC)—which is perhaps the most important determining factor when choosing an initial therapy. Tumor stage is classified according to the Tumor-Node-Metastasis staging system developed by a collaboration of the American Joint Commission on Cancer and Union for International Cancer Control, now in its 8 ^th^ edition which went into effect in January 2018 ^4^. Tumors categorized as NMIBC include carcinoma *in situ* (CIS), papillary (Ta), or invasive into or beyond the lamina propria (T1). Once the tumor invades the muscularis propria (T2) or beyond (T3 and T4), it is considered MIBC. Somewhat confusingly, these have been referred to as “superficial” and “invasive” disease, respectively, though that practice has been abandoned more recently for the sake of clarity. The tumor grade, describing the aggressiveness of tumor cells based on microscopic appearance, is also very important for the prognosis of bladder cancer. The 2016 World Health Organization classification system for urothelial carcinoma is divided into a binary system of high and low grade, though this is mainly applicable to Ta tumors, since nearly all (≥95%) disease ≥T1 is high grade and CIS is high grade by definition ^5^.

## Diagnosis

A complete clinical assessment for bladder cancer includes history, physical exam, imaging of the upper urinary tracts, and cystoscopy. Cystoscopy is the cornerstone in the diagnosis and treatment of bladder cancer, being used in virtually all patients at some point throughout the course of their disease. Traditionally, this is carried out using a standard light source (“white light”) to illuminate an endoscopic lens with the subsequent image transmitted via a camera head to a monitor. Directly visualized tumors are then biopsied, fulgurated, and/or resected to provide staging information that forms the basis for widely different treatments (i.e. surveillance versus radical cystectomy). Low-grade, papillary tumors not invading into the lamina propria (Ta) are reliably eradicated in a single setting; however, more advanced disease (high-grade and/or T1) is often incompletely resected. Studies have found residual disease to be present in 40–78% of re-transurethral resection (TUR) specimens after original diagnosis of high-grade Ta or any T1, with an upgrading rate to muscle invasion of 2% and 14%, respectively ^6– 9^. This fact has led to the recommendation by leading urologic organizations that all patients with T1 tumors should undergo a repeat resection within 6 weeks of the original procedure to confirm tumor stage and ensure maximal removal of any residual disease, as this improves response to intravesical therapy ^10, 11^. Recent technological advances, referred to as photodynamic aids (i.e. Blue Light® cystoscopy and narrow band imaging), promise improved detection over traditional white light cystoscopy alone, theoretically allowing for a more complete endoscopic tumor removal and more accurate risk assessment ^12– 19^.

The urothelium is not limited to the bladder and urethra; it also extends into the ureters and renal collecting system. The main purpose of imaging in the diagnostic evaluation of bladder cancer is to assess the upper urinary tracts for malignancy and for staging local and distant extent of disease. Computed tomographic urography, which uses intravenous contrast with delayed image acquisition (10–15 minutes) to allow for urinary excretion, has the highest diagnostic value for the detection of upper tract malignancy with a sensitivity of 67–100% and a specificity of 93–99% ^20– 23^. Magnetic resonance imaging is a viable alternative in patients with iodinated contrast allergy with a diagnostic accuracy of 84–92%, though it is more time intensive and still requires a contrast agent (gadolinium) and, therefore, is a poor choice in patients with significantly impaired renal function ^24^. Ultrasonography plus retrograde pyelography at the time of cystoscopy can be used if cross-sectional imaging is otherwise contraindicated, but this is suboptimal for assessing disease extent and upper tract involvement, so it is reserved for special circumstances. Upper tract evaluation is recommended as part of initial diagnostic work-up by the American Urological Association (AUA) and European Association of Urology (EAU) despite the very low likelihood of finding synchronous upper tract tumor at the time of NMIBC diagnosis (1.5%); however, certain features like multifocality, trigonal location, and CIS increase the risk (7.5%) ^10, 11, 25, 26^. A more nuanced approach to long-term surveillance is favored over repeat imaging, being applied only to patients with high-risk tumors, and is our first example of a risk-adapted approach to bladder cancer.

## Risk stratification in bladder cancer

Not all bladder cancers are created equal and, therefore, risk stratification is an important tool for achieving optimal patient outcomes while avoiding overtreatment. Formal classification systems exist for NMIBC given the wide variability in possible treatment options (surveillance to radical cystectomy), but a one-size-fits-all approach to MIBC is no longer appropriate either.

### Non-muscle-invasive bladder cancer

The risk tables from the European Organization for Research and Treatment of Cancer (EORTC) and scoring system from the Spanish Urological Club for Oncological Treatment (CUETO) for NMIBC classify patients into low, intermediate, or high risk for recurrence and progression to muscle invasion based on factors including grade, stage, tumor size, multifocality, variant histology, lymphovascular invasion, and prior therapy ^10, 11, 27, 28^. Validation studies based on these tools have shown consistent overestimation of recurrence and progression rates among the high-risk group, likely owing to the suboptimal administration of intravesical therapy seen in the developmental cohorts ^29– 32^. Though there is now widespread acceptance of induction intravesical immunotherapy (bacillus Calmette-Guérin [BCG]) plus maintenance therapy for 1 to 3 years based on the results of the randomized Southwest Oncology Group (SWOG) protocol, patients from EORTC and CUETO were largely treated with intravesical chemotherapies (mitomycin C, epirubicin, thiotepa, etc.) or a lack of appropriate maintenance BCG ^33^. Despite the limitations of both aforementioned studies, they form the foundation for the risk stratification systems used by the EAU and AUA to help guide treatment decisions ( Table 1) ^10, 11, 34^.

**Table 1.** Risk stratification of non-muscle-invasive bladder cancer ^10, 11^.

Risk category	EAU definition ^9^	EAU recommendations	AUA definition ^10^	AUA recommendations
Low	• Primary, • Solitary (<3 cm), • Low-grade/G1, and • Ta	• Single immediate post-TUR instillation of chemotherapy	• Solitary <3 cm, • Low-grade, and • Ta	• Single immediate post-TUR instillation of chemotherapy
Intermediate	• Any disease not fitting low- or high-risk criteria	• Single immediate post-TUR instillation of chemotherapy, and either • Induction chemotherapy for 1 year, or • Induction BCG with 1 year of maintenance therapy	• Low-grade Ta recurrence <1 year • Solitary low-grade Ta >3 cm, • Multifocal low-grade Ta, • High-grade Ta ≤3 cm, or • Low-grade T1	• Single immediate post-TUR instillation of chemotherapy, and either • Induction chemotherapy with or without maintenance, or • Induction BCG with maintenance
High	• Any T1, or • High-grade/G3, or • CIS present, or • Multiple, recurrent, large (>3 cm), papillary (Ta), low-grade/G1 or G2 tumors	• Single immediate post-TUR instillation of chemotherapy, and either • Induction BCG with 1–3 years of maintenance therapy, or • Immediate radical cystectomy	• High-grade T1 • Recurrent high-grade Ta • High-grade Ta >3 cm • CIS • Any high-grade failing BCG • Variant histology • LVI • High-grade prostatic urethral involvement	• Induction BCG with maintenance therapy, or • Immediate radical cystectomy for highest risk features (with LVI, variant histology, T1 with CIS, persistent T1 on re-TUR)

AUA, American Urological Association; BCG, bacillus Calmette-Guérin; CIS, carcinoma *in situ*; EAU, European Association of Urology; LVI, lymphovascular invasion; TUR, transurethral resection

**Figure 1.**
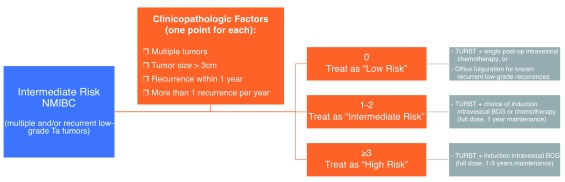
Proposed substratification of intermediate-risk non-muscle-invasive bladder cancer (NMIBC) based on the recommendations of the International Bladder Cancer Group ^35^. BCG, bacillus Calmette-Guérin; TURBT, transurethral resection of bladder tumor.

There is general agreement among urological organizations that a more-conservative approach to treatment (cystoscopic resection and single-dose intravesical chemotherapy) is warranted for low-risk tumors (solitary, low-grade, papillary), while the high-risk group (multifocal high-grade, CIS, or any T1) should be managed aggressively through the use of intravesical immunotherapy, and even radical cystectomy in some cases ^10, 11^. This leaves a broad middle ground for intermediate-risk disease, representing a spectrum ranging from a small, recurrent low-grade papillary tumor to large treatment-resistant low-grade tumors to small high-grade lesions. In order to more effectively tailor an appropriate treatment regimen for these patients, further substratification of intermediate-risk bladder cancer has been proposed by an international consortium of bladder cancer experts using a simple scoring system based on four main tumor features ( Figure 1) ^35^.

### Muscle-invasive bladder cancer

MIBC is often treated as a single disease entity with only one acceptable therapy in the form of neoadjuvant chemotherapy followed by radical cystectomy with urinary diversion and lymphadenectomy ^36^. However, risk assessment can be applied before or after definitive therapy to identify which patients require the most aggressive approach, specifically those that include adjuvant treatments with significant added toxicity. For example, the decision to delay radical cystectomy so that neoadjuvant chemotherapy can be administered for a small—5% absolute improvement in overall survival at 5 years—but statistically significant benefit can be more precisely applied only to patients with the highest chance of seeing a benefit ^37^. This topic will be re-addressed in more detail in a later section. Although lacking consensus recommendations for risk grouping from any major urological societies, the stratification and personalization of MIBC therapy is on the verge of a significant paradigm shift supported by a growing body of genomic data.

## Pathologic subtypes

The most common histology of bladder cancer is urothelial carcinoma (~90%) followed by squamous, adenocarcinoma, micropapillary, small cell, and other rare tumors ^38^. Not all urothelial carcinoma exists in pure form; instead divergent differentiation may occur within the tumor exhibiting different morphology and labeled as “variant histology” ^5^. Of these variants, several are worth noting, as certain subtypes are known to be associated with a more aggressive clinical course. When taken as a group, variant histology is associated with more advanced stage at diagnosis and may not be amenable to standard treatment algorithms, even when adjusting for stage. For instance, plasmacytoid is particularly aggressive with up to 90% diagnosed with extension outside of the bladder (≥T3) and poor responsiveness to cisplatinum-based adjuvant chemotherapy following radical cystectomy ^39– 41^. Early cystectomy appears to offer survival advantage for micropapillary disease, even for clinical stage T1, because of a high rate of pathologic upstaging and node positivity ^42– 46^. Squamous differentiation, the most recognized subtype, and glandular are often reported on pathology reports; however, clinical outcomes are no different than those for conventional urothelial carcinoma ^47, 48^. These should also be recognized as very different to pure squamous cell carcinoma and adenocarcinoma of the bladder, which are distinct histologic entities lacking urothelial components ^49, 50^. Other less-common variants worth mentioning include nested, lymphoepithelioma-like, and small cell, the latter being morphologically similar to the lung cancer version and sharing the same aggressive clinical course ^51, 52^.

## Molecular subtyping

MIBC ranks among the most highly mutated cancers, with frequencies similar to non-small cell lung cancers and head and neck squamous cell carcinoma, resulting in a heterogeneous mutational profile involving numerous cellular pathways ^53– 56^. Investigators from around the world have taken on the task of genetically characterizing MIBC samples, resulting in the publication of several major molecular classification systems (the Cancer Genome Atlas [TCGA], Lund, University of North Carolina, MD Anderson, etc.) ^55, 57– 59^. Each analysis yielded groups of tumors enriched with certain mutations that shared common features with other carcinomas, specifically the breast cancer “basal” and “luminal” subtypes, which led to similarities in the naming schemes. Broadly speaking, basal tumors tend to exhibit a more aggressive phenotype than do luminal tumors and are more prone to metastasis at the time of diagnosis ^55– 60^. The major publications on this topic have each included a classification system with more-or-less overlap in the mutational profile—i.e. basal-like (UNC), UroB (Lund), Cluster III (TCGA), etc.—that correlates with clinical outcomes. An effort to achieve consensus definitions for all subtypes is underway, thus far producing the Basal-Squamous-like group (BASQ) with elevated *KRT5/6* and *KRT14* expression but low *FOXA1* and *GATA3* expression ^61^. By using the information from cohorts like TCGA, several researchers have sought to define the prognostic significance of these genetic mutations through the creation of molecular subtypes with correlation with clinical outcomes ^56– 58, 62, 63^.

**Figure 2.**
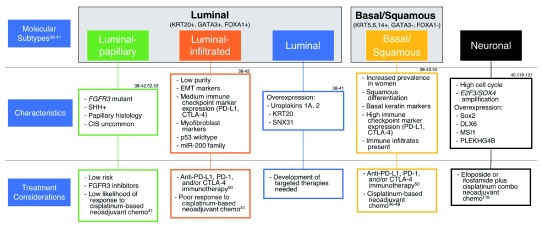
Molecular subtypes of muscle-invasive bladder cancer with associated clinicopathologic and genomic characteristics. Proposed treatment considerations are listed for each subtype based on available clinical data ^53– 57, 63, 64, 66– 69^. CIS, carcinoma *in situ*; CTLA-4, cytotoxic T-lymphocyte-associated antigen 4; DLX6, distal-less homeobox 6; E2F3, E2F transcription factor 3; EMT, epithelial to mesenchymal transition; FGFR3, fibroblast growth factor receptor 3; FOX, forkhead box; GATA, GATA-binding protein; KRT, keratin; miR-200, microRNA 200; msi1, Musashi homolog 1; PD-1, programmed death-1; PD-L1, programmed death-ligand 1; PLEKHG4B, pleckstrin homology and RhoGEF domain containing G4B; SHH, sonic hedgehog; SNX31, sorting nexin 31; SOX, SRY-box.

The association of molecular subtypes with response to bladder cancer therapy is certain to help guide treatment in the near future: for instance, which patients are most likely to benefit from neoadjuvant chemotherapy prior to surgery or response to immunotherapy over conventional chemotherapy ^56, 57, 64^. Systemic therapy for bladder cancer can then be more carefully tailored to the individual patient using information obtained from genetic sequencing to select the best candidates for a given treatment regimen ( Figure 2). For example, tumors exhibiting mutations in genes associated with DNA damage repair (i.e. *ERCC2*, *ERBB2*, *ATM*, and *RB1*) or those grouped into the basal/squamous subtype display a greater degree of cisplatinum sensitivity as demonstrated by more favorable clinical outcomes among these patients ^56, 57, 65– 68^. The luminal-papillary and luminal-infiltrated subtypes, on the other hand, show poor response to neoadjuvant chemotherapy; however, tumors with upregulation of the immune checkpoint markers (luminal-infiltrated and basal/squamous) may benefit from treatment with anti-PD-L1, PD-1, and/or CTLA-4 immunotherapy ^56, 69^.

The clinical impact of NMIBC is just as significant as that of MIBC, though maybe not as immediate a threat to survival, especially when taking into account rates of progression reaching as high as 50–60%. A considerable amount of information is available on molecular subtyping for NMIBC that is helping researchers hone in on the most important mutations in the evolution of bladder cancer ^59, 70, 71^. Low-grade bladder tumors would appear to be genetically distinct from high-grade and MIBC, demonstrating highly conserved genetic mutations of *FGFR3*, *PIK3CA*, *STAG2*, and/or the RTK/RAS/RAF pathway ^72– 75^. High-grade NMIBC, on the other hand, is more like muscle-invasive disease, exhibiting alteration of DNA damage repair genes ( *ERBB2*), cell cycle regulators ( *p53*, *RB1*, *MDM2*, and *CDKN2A*), and chromatin-modifying genes ( *KDM6A* and *ARID1A*) ^75^. Molecular subtyping based on transcriptome analysis of 460 NMIBC and 16 MIBC samples by Hedegaard *et al*. identified three predominant classes of NMIBC, of which class two showed clustering of high-grade and CIS pathology with an increased risk of clinical progression, indicating that this may be a signature warranting more aggressive, definitive therapy upfront ^71^. The *TERT* promoter is worth specific attention owing to similar frequency throughout all tumor stages and grades, regardless of tumor aggressiveness or molecular subtype, and likely represents an early event in the development of urothelial neoplasms ^76– 78^.

## Personalized approach to bladder cancer therapy

### Intravesical therapies

Instillation of chemotherapy immediately (within 24 hours) following TUR has been proposed as a method of reducing bladder cancer recurrences, specifically for low- and intermediate-risk disease, and is recommended by both the AUA and the EAU guidelines for NMIBC ^10, 11^. It is hypothesized to work via two main methods: (1) residual tumor that has not been fully resected will be killed and (2) elimination of free-floating cancerous cells dislodged during resection that may adhere to the urothelium and proliferate in a region separate from the primary ^79, 80^. The most well-studied agents include mitomycin C, doxorubicin, and epirubicin, all of which have been found to reduce recurrences (HR 0.40–0.65) without impacting progression or survival outcomes ^81– 89^. This is probably because of the preponderance of low-risk tumors included in the study cohorts, which are already at very low risk of progression to begin with. According to a recent, large meta-analysis, immediate post-resection instillation would appear to be most effective for small (<3 cm), low-grade, papillary tumors with a baseline recurrence rate of >1 year ^89^. Not all urologists support this mode of treatment owing to the small but catastrophic possibility of chemotherapy leaking into the perivesical space via missed bladder perforation, in some cases resulting in end-stage bladder fibrosis, for the minimal clinical benefit of prolonging time between recurrences of relatively indolent tumors ^90, 91^. Though the overwhelming majority of available literature may offer support for this viewpoint, Bosscheiter *et al*. published a prospective, randomized trial of immediate post-operative mitomycin C versus instillation 2 weeks later among more than 2,000 patients across all risk categories, finding an overall reduction in 3-year recurrence (27% versus 36%) and progression (2.7% versus 5.5%) ^92^. Interestingly, on subgroup analysis, only the intermediate- and high-risk patients—who also received an adjuvant 6- to 12-week course of intravesical mitomycin C—were found to benefit from the immediate post-operative dose.

Induction intravesical therapy refers to a period of weekly instillations of chemotherapy (i.e. mitomycin C, epirubicin, gemcitabine, etc.) or immunotherapy (i.e. BCG) following a complete TUR for NMIBC. At this point, TUR followed by a 6-week course of BCG plus 1 to 3 years of appropriate (3-weekly instillations based on SWOG protocol) maintenance therapy is the first-line option for high-risk disease with a proven improvement in disease recurrence and progression ^10, 11, 93– 96^. Intermediate-risk bladder cancer, on the other hand, can be successfully treated with either chemotherapy or immunotherapy; however, BCG must be followed by at least 1 year of maintenance in order to maintain an advantage in recurrence over mitomycin C ^94, 97^. Furthermore, while only BCG has shown improvement in progression for these patients, the potential side effects should be considered when selecting a treatment strategy ^97– 99^.

Patients who fail an adequate induction course of intravesical therapy, particularly those receiving BCG who are at highest risk for disease progression, pose a therapeutic dilemma: remove the bladder immediately to prevent muscle invasion and possible metastasis or continue local treatment with further instillations. It is preferable to avoid the morbidity and impact on quality of life associated with radical cystectomy; however, there are currently few alternatives in this setting. The lack of a standardized definition of BCG failure, recognizing that not all forms share the same prognosis, has hindered research into this area. To address these shortcomings, an international panel published guidance for clinicians and researchers to aid in creating more uniformity when designing trials and reporting on this group of patients, as well as to differentiate those with poorest prognosis (BCG unresponsive) who may not benefit from further BCG therapy ( Table 2) ^100^. The AUA recommends enrolment in clinical trials for patients who have demonstrated BCG unresponsiveness but are unwilling or unable to undergo a radical cystectomy ^11^. Several such trials have offered promising results, with most reporting short-term reduction in recurrence of approximately 30–50%, but longer follow-up is associated with sharp declines in responsiveness, and, therefore, there is insufficient evidence to support any single approach, and radical cystectomy remains the gold standard in this population ^94, 101– 107^.

**Table 2.** A classification system for bacillus Calmette-Guérin (BCG) failures ^100^.

Type of failure	Description
BCG refractory	Persistence of high-grade disease at 6 months (3 months for T1 high grade) following adequate BCG treatment
BCG relapsing	Recurrence of high-grade disease following adequate BCG treatment with a disease-free period of 6 months
BCG unresponsive	Includes BCG refractory and BCG relapses within 6 months (12 months for carcinoma *in situ* patients)
BCG intolerant	Persistence of high-grade disease in a patient who is unable to tolerate induction BCG secondary to toxicity

### Early cystectomy for non-muscle-invasive bladder cancer

**Figure 3.**
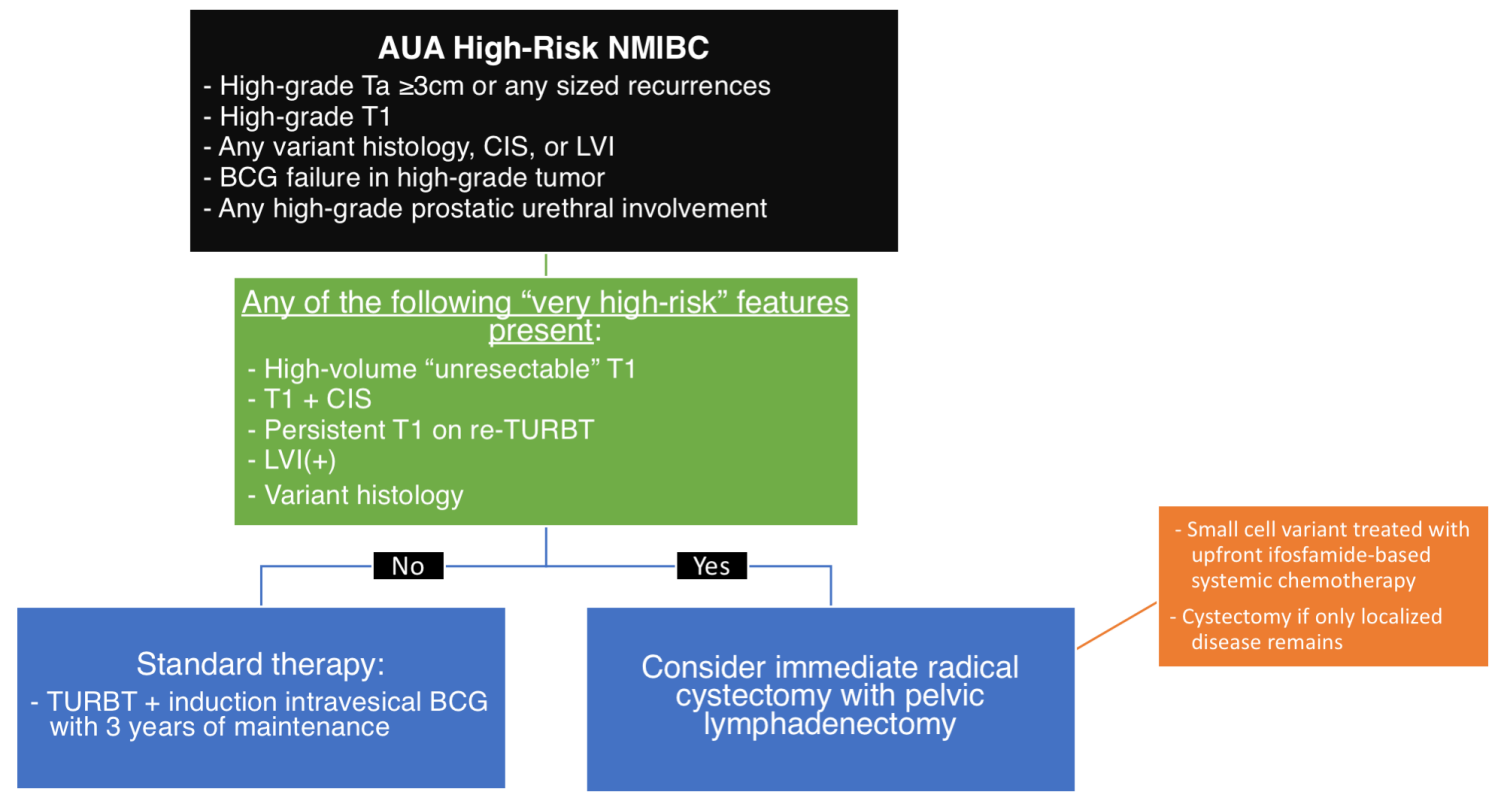
Proposed decision model for immediate radical cystectomy in patients with “very high-risk” non-muscle-invasive bladder cancer (NMIBC) ^11, 125– 129^. AUA, American Urological Association; BCG, bacillus Calmette-Guérin; CIS, carcinoma *in situ*; LVI, lymphovascular invasion; TURBT, transurethral resection of bladder tumor.

Immediate radical cystectomy may be the best treatment option for selected patients with certain adverse “very high-risk” factors found at the time of diagnosis of NMIBC ( Figure 3). High-volume T1, persistent high-grade T1 on re-TUR, lymphovascular invasion, certain variant histologies (i.e. micropapillary, plasmacytoid, nested), or concomitant CIS have all been associated with increased risk of progression and are best managed with surgery ^108– 113^. A group from the United Kingdom has designed a prospective, randomized controlled trial, with end of accrual set for March 2018, comparing immediate cystectomy against BCG induction with maintenance therapy for a “very high-risk” population of NMIBC patients ^114^. Notably excluded from their cohort are any patients with variant histology.

### Radiotherapy

Trimodal bladder-preserving therapy involves complete endoscopic resection of all visible tumor followed by neoadjuvant chemotherapy and definitive whole-bladder external beam radiotherapy. The use of this strategy is not widespread in the United States, but prospective European cohorts have demonstrated comparable disease-specific outcomes when compared to contemporary radical cystectomy cohorts ^115^. Limited evidence regarding optimal patient selection has identified increased expression of MRE11, a protein involved in the cellular response to radiation damage, as a potential prognostic marker for improved cancer-specific survival following radiotherapy ^116, 117^.

### Timing of chemotherapy for muscle-invasive bladder cancer

**Figure 4.**
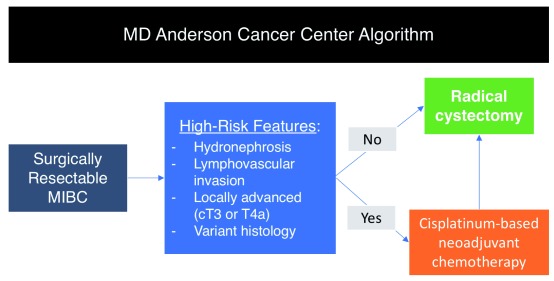
Current MD Anderson Cancer Center algorithm for determining which patients should receive neoadjuvant chemotherapy versus immediate radical cystectomy. Inclusion of molecular markers for further risk stratification is pending clinical validation ^37^. MIBC, muscle-invasive bladder cancer.

Muscle invasion at diagnosis of bladder cancer is a poor prognostic indicator best treated with neoadjuvant cisplatinum-based multi-agent chemotherapy followed by radical cystectomy according to evidence from several phase III clinical trials ^118– 121^. Disease status on final pathology is strongly correlated with oncologic outcomes, and neoadjuvant therapy leads to significant downstaging, including rates of complete response in the range of 30–40% compared to only 15% with surgery alone ^119^. However, this still leaves a large portion of patients who will not derive benefit, instead undergoing unnecessary toxic therapy while at the same time delaying surgery. Risk stratification can be useful in this setting by selecting those patients with the highest risk (pre-operative T3b–T4, hydroureteronephrosis, lymphovascular invasion, and specific histologic variants) for poor outcomes after cystectomy and only administering neoadjuvant chemotherapy to this subgroup ( Figure 4) ^122, 123^. Likewise, post-operative chemotherapy (i.e. adjuvant) can be administered in patients with proven pathologic predictors of developing metastasis, though evidence to support its use is lacking. One study (EORTC 30994) was able to show an improvement in progression-free survival; however, there was no statistically significant impact on overall survival ^124^.

## Conclusions

The appropriate management of bladder cancer patients relies heavily upon risk stratification to personalize the therapy for the patient. Currently, clinicopathologic features are the cornerstone of the most widely used risk assessment tools with ample room for improvement. Advances in genetic sequencing of tumors has led to classification systems based on the mutational profile of each individual, offering prognostic information. As these data expand, it is feasible that in the near future this could be integrated to provide guidance that is truly “next generation”.

## Abbreviations

AUA, American Urological Association; BCG, bacillus Calmette-Guérin; CIS, carcinoma *in situ*; CUETO, Spanish Urological Club for Oncological Treatment; EAU, European Association of Urology; EORTC, European Organization for Research and Treatment of Cancer; MIBC, muscle-invasive bladder cancer; NMIBC, non-muscle-invasive bladder cancer; SWOG, Southwest Oncology Group; TCGA, the Cancer Genome Atlas; TUR, transurethral resection

## References

[1] Burger M Catto JW Dalbagni G: Epidemiology and risk factors of urothelial bladder cancer. *Eur Urol.* 2013;63(2):234–41. 10.1016/j.eururo.2012.07.033 22877502

[2] Siegel RL Miller KD Jemal A: Cancer statistics, 2018. *CA Cancer J Clin.* 2018;68(1):7–30. 10.3322/caac.21442 29313949

[3] Torre LA Bray F Siegel RL: Global cancer statistics, 2012. *CA Cancer J Clin.* 2015;65(2):87–108. 10.3322/caac.21262 25651787

[4] American Joint Committee on Cancer: AJCC cancer staging manual. Eighth edition. Amin MB, Edge SB, ed. Switzerland: Springer;2017 Reference Source

[5] Humphrey PA Moch H Cubilla AL: The 2016 WHO Classification of Tumours of the Urinary System and Male Genital Organs-Part B: Prostate and Bladder Tumours. *Eur Urol.* 2016;70(1):106–19. 10.1016/j.eururo.2016.02.028 26996659

[6] Divrik RT Yildirim U Zorlu F: The effect of repeat transurethral resection on recurrence and progression rates in patients with T1 tumors of the bladder who received intravesical mitomycin: a prospective, randomized clinical trial. *J Urol.* 2006;175(5):1641–4. 10.1016/S0022-5347(05)01002-5 16600720

[7] Gendy R Delprado W Brenner P: Repeat transurethral resection for non-muscle-invasive bladder cancer: a contemporary series. *BJU Int.* 2016;117 Suppl 4:54–9. 10.1111/bju.13265 26486968

[8] Lazica DA Roth S Brandt AS: Second transurethral resection after Ta high-grade bladder tumor: a 4.5-year period at a single university center. *Urol Int.* 2014;92(2):131–5. 10.1159/000353089 23988813

[9] Cumberbatch MGK Foerster B Catto JWF: Repeat Transurethral Resection in Non-muscle-invasive Bladder Cancer: A Systematic Review. *Eur Urol.* 2018;73(6):925–33. 10.1016/j.eururo.2018.02.014 29523366

[10] Babjuk M Böhle A Burger M: EAU Guidelines on Non-Muscle-invasive Urothelial Carcinoma of the Bladder: Update 2016. *Eur Urol.* 2017;71(3):447–61. 10.1016/j.eururo.2016.05.041 27324428

[11] Chang SS Boorjian SA Chou R: Diagnosis and Treatment of Non-Muscle Invasive Bladder Cancer: AUA/SUO Guideline. *J Urol.* 2016;196(4):1021–9. 10.1016/j.juro.2016.06.049 27317986

[12] Burger M Grossman HB Droller M: Photodynamic diagnosis of non-muscle-invasive bladder cancer with hexaminolevulinate cystoscopy: a meta-analysis of detection and recurrence based on raw data. *Eur Urol.* 2013;64(5):846–54. 10.1016/j.eururo.2013.03.059 23602406

[13] Chang TC Marcq G Kiss B: Image-Guided Transurethral Resection of Bladder Tumors - Current Practice and Future Outlooks. *Bladder Cancer.* 2017;3(3):149–59. 10.3233/BLC-170119 28824942 PMC5545914

[14] Denzinger S Burger M Walter B: Clinically relevant reduction in risk of recurrence of superficial bladder cancer using 5-aminolevulinic acid-induced fluorescence diagnosis: 8-year results of prospective randomized study. *Urology.* 2007;69(4):675–9. 10.1016/j.urology.2006.12.023 17445650

[15] Herr HH: Narrow band imaging cystoscopy. *Urol Oncol.* 2011;29(4):353–7. 10.1016/j.urolonc.2010.12.002 21858935

[16] Kang W Cui Z Chen Q: Narrow band imaging-assisted transurethral resection reduces the recurrence risk of non-muscle invasive bladder cancer: A systematic review and meta-analysis. *Oncotarget.* 2017;8(14):23880–90. 10.18632/oncotarget.13054 27823975 PMC5410352

[17] Lee JY Cho KS Kang DH: A network meta-analysis of therapeutic outcomes after new image technology-assisted transurethral resection for non-muscle invasive bladder cancer: 5-aminolaevulinic acid fluorescence vs hexylaminolevulinate fluorescence vs narrow band imaging. *BMC Cancer.* 2015;15:566. 10.1186/s12885-015-1571-8 26232037 PMC4521364

[18] Rink M Babjuk M Catto JW: Hexyl aminolevulinate-guided fluorescence cystoscopy in the diagnosis and follow-up of patients with non-muscle-invasive bladder cancer: a critical review of the current literature. *Eur Urol.* 2013;64(4):624–38. 10.1016/j.eururo.2013.07.007 23906669

[19] Rolevich AI Zhegalik AG Mokhort AA: Results of a prospective randomized study assessing the efficacy of fluorescent cystoscopy-assisted transurethral resection and single instillation of doxorubicin in patients with non-muscle-invasive bladder cancer. *World J Urol.* 2017;35(5):745–52. 10.1007/s00345-016-1927-y 27604374

[20] Chlapoutakis K Theocharopoulos N Yarmenitis S: Performance of computed tomographic urography in diagnosis of upper urinary tract urothelial carcinoma, in patients presenting with hematuria: Systematic review and meta-analysis. *Eur J Radiol.* 2010;73(2):334–8. 10.1016/j.ejrad.2008.10.026 19058939

[21] Cowan NC Turney BW Taylor NJ: Multidetector computed tomography urography for diagnosing upper urinary tract urothelial tumour. *BJU Int.* 2007;99(6):1363–70. 10.1111/j.1464-410X.2007.06766.x 17428251

[22] Froemming A Potretzke T Takahashi N: Upper tract urothelial cancer. *Eur J Radiol.* 2018;98:50–60. 10.1016/j.ejrad.2017.10.021 29279170

[23] Rouprêt M Babjuk M Compérat E: European Association of Urology Guidelines on Upper Urinary Tract Urothelial Carcinoma: 2017 Update. *Eur Urol.* 2018;73(1):111–22. 10.1016/j.eururo.2017.07.036 28867446

[24] Takahashi N Glockner JF Hartman RP: Gadolinium enhanced magnetic resonance urography for upper urinary tract malignancy. *J Urol.* 2010;183(4):1330–65. 10.1016/j.juro.2009.12.031 20171676

[25] Palou J Rodríguez-Rubio F Huguet J: Multivariate analysis of clinical parameters of synchronous primary superficial bladder cancer and upper urinary tract tumor. *J Urol.* 2005;174(3):859–61; discussion 861. 10.1097/01.ju.0000169424.79702.6d 16093970

[26] Millán-Rodríguez F Chéchile-Toniolo G Salvador-Bayarri J: Upper urinary tract tumors after primary superficial bladder tumors: prognostic factors and risk groups. *J Urol.* 2000;164(4):1183–7. 10.1016/S0022-5347(05)67137-6 10992362

[27] Fernandez-Gomez J Madero R Solsona E: Predicting nonmuscle invasive bladder cancer recurrence and progression in patients treated with bacillus Calmette-Guerin: the CUETO scoring model. *J Urol.* 2009;182(5):2195–203. 10.1016/j.juro.2009.07.016 19758621

[28] Sylvester RJ van der Meijden AP Oosterlinck W: Predicting recurrence and progression in individual patients with stage Ta T1 bladder cancer using EORTC risk tables: a combined analysis of 2596 patients from seven EORTC trials. *Eur Urol.* 2006;49(3):466–5; discussion 475–7. 10.1016/j.eururo.2005.12.031 16442208

[29] Fernandez-Gomez J Madero R Solsona E: The EORTC tables overestimate the risk of recurrence and progression in patients with non-muscle-invasive bladder cancer treated with bacillus Calmette-Guérin: external validation of the EORTC risk tables. *Eur Urol.* 2011;60(3):423–30. 10.1016/j.eururo.2011.05.033 21621906

[30] Hernández V De La Peña E Martin MD: External validation and applicability of the EORTC risk tables for non-muscle-invasive bladder cancer. *World J Urol.* 2011;29(4):409–14. 10.1007/s00345-010-0635-2 21190023

[31] Seo KW Kim BH Park CH: The efficacy of the EORTC scoring system and risk tables for the prediction of recurrence and progression of non-muscle-invasive bladder cancer after intravesical bacillus calmette-guerin instillation. *Korean J Urol.* 2010;51(3):165–70. 10.4111/kju.2010.51.3.165 20414391 PMC2855454

[32] Xylinas E Kent M Kluth L: Accuracy of the EORTC risk tables and of the CUETO scoring model to predict outcomes in non-muscle-invasive urothelial carcinoma of the bladder. *Br J Cancer.* 2013;109(6):1460–6. 10.1038/bjc.2013.372 23982601 PMC3776972

[33] Lamm DL Blumenstein BA Crissman JD: Maintenance bacillus Calmette-Guerin immunotherapy for recurrent TA, T1 and carcinoma *in situ* transitional cell carcinoma of the bladder: a randomized Southwest Oncology Group Study. *J Urol.* 2000;163(4):1124–9. 10.1016/S0022-5347(05)67707-5 10737480

[34] van Kessel KEM van der Keur KA Dyrskjøt L: Molecular Markers Increase Precision of the European Association of Urology Non-Muscle-Invasive Bladder Cancer Progression Risk Groups. *Clin Cancer Res.* 2018;24(7):1586–93. 10.1158/1078-0432.CCR-17-2719 29367430

[35] Kamat AM Witjes JA Brausi M: Defining and treating the spectrum of intermediate risk nonmuscle invasive bladder cancer. *J Urol.* 2014;192(2):305–15. 10.1016/j.juro.2014.02.2573 24681333 PMC4687397

[36] Chang SS Bochner BH Chou R: Treatment of Non-Metastatic Muscle-Invasive Bladder Cancer: AUA/ASCO/ASTRO/SUO Guideline. *J Urol.* 2017;198(3):552–9. 10.1016/j.juro.2017.04.086 28456635 PMC5626446

[37] Karam JA Kamat AM: Optimal timing of chemotherapy and cystectomy. *F1000 Med Rep.* 2010;2: pii: 48. 10.3410/M2-48 20948836 PMC2950042

[38] Hansel DE Amin MB Comperat E: A contemporary update on pathology standards for bladder cancer: transurethral resection and radical cystectomy specimens. *Eur Urol.* 2013;63(2):321–32. 10.1016/j.eururo.2012.10.008 23088996

[39] Kaimakliotis HZ Monn MF Cary KC: Plasmacytoid variant urothelial bladder cancer: is it time to update the treatment paradigm? *Urol Oncol.* 2014;32(6):833–8. 10.1016/j.urolonc.2014.03.008 24954925

[40] Keck B Stoehr R Wach S: The plasmacytoid carcinoma of the bladder--rare variant of aggressive urothelial carcinoma. *Int J Cancer.* 2011;129(2):346–54. 10.1002/ijc.25700 20878954

[41] Keck B Wach S Stoehr R: Plasmacytoid variant of bladder cancer defines patients with poor prognosis if treated with cystectomy and adjuvant cisplatin-based chemotherapy. *BMC Cancer.* 2013;13:71. 10.1186/1471-2407-13-71 23394492 PMC3572418

[42] Fernández MI Williams SB Willis DL: Clinical risk stratification in patients with surgically resectable micropapillary bladder cancer. *BJU Int.* 2017;119(5):684–91. 10.1111/bju.13689 27753185

[43] Kamat AM Dinney CP Gee JR: Micropapillary bladder cancer: a review of the University of Texas M. D. Anderson Cancer Center experience with 100 consecutive patients. *Cancer.* 2007;110(1):62–7. 10.1002/cncr.22756 17542024

[44] Meeks JJ Taylor JM Matsushita K: Pathological response to neoadjuvant chemotherapy for muscle-invasive micropapillary bladder cancer. *BJU Int.* 2013;111(8):E325–30. 10.1111/j.1464-410X.2012.11751.x 23384236

[45] Sui W Matulay JT James MB: Micropapillary Bladder Cancer: Insights from the National Cancer Database. *Bladder Cancer.* 2016;2(4):415–23. 10.3233/BLC-160066 28035322 PMC5181670

[46] Vetterlein MW Wankowicz SAM Seisen T: Neoadjuvant chemotherapy prior to radical cystectomy for muscle-invasive bladder cancer with variant histology. *Cancer.* 2017;123(22):4346–55. 10.1002/cncr.30907 28743155

[47] Amin MB: Histological variants of urothelial carcinoma: diagnostic, therapeutic and prognostic implications. *Mod Pathol.* 2009;22 Suppl 2:S96–S118. 10.1038/modpathol.2009.26 19494856

[48] Mitra AP Bartsch CC Bartsch G Jr: Does presence of squamous and glandular differentiation in urothelial carcinoma of the bladder at cystectomy portend poor prognosis? An intensive case-control analysis. *Urol Oncol.* 2014;32(2):117–27. 10.1016/j.urolonc.2012.08.017 23477878

[49] Abdollah F Sun M Jeldres C: Survival after radical cystectomy of non-bilharzial squamous cell carcinoma vs urothelial carcinoma: a competing-risks analysis. *BJU Int.* 2012;109(4):564–9. 10.1111/j.1464-410X.2011.10357.x 21810161

[50] Zaghloul MS Nouh A Nazmy M: Long-term results of primary adenocarcinoma of the urinary bladder: a report on 192 patients. *Urol Oncol.* 2006;24(1):13–20. 10.1016/j.urolonc.2005.05.027 16414487

[51] Warrick JI: Clinical Significance of Histologic Variants of Bladder Cancer. *J Natl Compr Canc Netw.* 2017;15(10):1268–74. 10.6004/jnccn.2017.7027 28982751

[52] Lynch SP Shen Y Kamat A: Neoadjuvant chemotherapy in small cell urothelial cancer improves pathologic downstaging and long-term outcomes: results from a retrospective study at the MD Anderson Cancer Center. *Eur Urol.* 2013;64(2):307–13. 10.1016/j.eururo.2012.04.020 22564397 PMC3815632

[53] Kandoth C McLellan MD Vandin F: Mutational landscape and significance across 12 major cancer types. *Nature.* 2013;502(7471):333–9. 10.1038/nature12634 24132290 PMC3927368

[54] Yap KL Kiyotani K Tamura K: Whole-exome sequencing of muscle-invasive bladder cancer identifies recurrent mutations of *UNC5C* and prognostic importance of DNA repair gene mutations on survival. *Clin Cancer Res.* 2014;20(24):6605–17. 10.1158/1078-0432.CCR-14-0257 25316812 PMC4268280

[55] Cancer Genome Atlas Research Network: Comprehensive molecular characterization of urothelial bladder carcinoma. *Nature.* 2014;507(7492):315–22. 10.1038/nature12965 24476821 PMC3962515

[56] Robertson AG Kim J Al-Ahmadie H: Comprehensive Molecular Characterization of Muscle-Invasive Bladder Cancer. *Cell.* 2017;171(3):540–556.e25. 10.1016/j.cell.2017.09.007 28988769 PMC5687509

[57] Choi W Porten S Kim S: Identification of distinct basal and luminal subtypes of muscle-invasive bladder cancer with different sensitivities to frontline chemotherapy. *Cancer Cell.* 2014;25(2):152–65. 10.1016/j.ccr.2014.01.009 24525232 PMC4011497

[58] Damrauer JS Hoadley KA Chism DD: Intrinsic subtypes of high-grade bladder cancer reflect the hallmarks of breast cancer biology. *Proc Natl Acad Sci U S A.* 2014;111(8):3110–5. 10.1073/pnas.1318376111 24520177 PMC3939870

[59] Lindgren D Frigyesi A Gudjonsson S: Combined gene expression and genomic profiling define two intrinsic molecular subtypes of urothelial carcinoma and gene signatures for molecular grading and outcome. *Cancer Res.* 2010;70(9):3463–72. 10.1158/0008-5472.CAN-09-4213 20406976

[60] Kamat AM Hahn NM Efstathiou JA: Bladder cancer. *Lancet.* 2016;388(10061):2796–810. 10.1016/S0140-6736(16)30512-8 27345655

[61] Lerner SP McConkey DJ Hoadley KA: Bladder Cancer Molecular Taxonomy: Summary from a Consensus Meeting. *Bladder Cancer.* 2016;2(1):37–47. 10.3233/BLC-150037 27376123 PMC4927916

[62] Rebouissou S Bernard-Pierrot I de Reyniès A: EGFR as a potential therapeutic target for a subset of muscle-invasive bladder cancers presenting a basal-like phenotype. *Sci Transl Med.* 2014;6(244):244ra91. 10.1126/scitranslmed.3008970 25009231

[63] Sjödahl G Eriksson P Liedberg F: Molecular classification of urothelial carcinoma: global mRNA classification versus tumour-cell phenotype classification. *J Pathol.* 2017;242(1):113–25. 10.1002/path.4886 28195647 PMC5413843

[64] Seiler R Ashab HAD Erho N: Impact of Molecular Subtypes in Muscle-invasive Bladder Cancer on Predicting Response and Survival after Neoadjuvant Chemotherapy. *Eur Urol.* 2017;72(4):544–54. 10.1016/j.eururo.2017.03.030 28390739

[65] Groenendijk FH de Jong J Fransen van de Putte EE: *ERBB2* Mutations Characterize a Subgroup of Muscle-invasive Bladder Cancers with Excellent Response to Neoadjuvant Chemotherapy. *Eur Urol.* 2016;69(3):384–8. 10.1016/j.eururo.2015.01.014 25636205

[66] Liu D Plimack ER Hoffman-Censits J: Clinical Validation of Chemotherapy Response Biomarker *ERCC2* in Muscle-Invasive Urothelial Bladder Carcinoma. *JAMA Oncol.* 2016;2(8):1094–6. 10.1001/jamaoncol.2016.1056 27310333 PMC5515075

[67] Plimack ER Dunbrack RL Brennan TA: Defects in DNA Repair Genes Predict Response to Neoadjuvant Cisplatin-based Chemotherapy in Muscle-invasive Bladder Cancer. *Eur Urol.* 2015;68(6):959–67. 10.1016/j.eururo.2015.07.009 26238431 PMC4764095

[68] van Allen EM Mouw KW Kim P: Somatic *ERCC2* mutations correlate with cisplatin sensitivity in muscle-invasive urothelial carcinoma. *Cancer Discov.* 2014;4(10):1140–53. 10.1158/2159-8290.CD-14-0623 25096233 PMC4238969

[69] Ren R Tyryshkin K Graham CH: Comprehensive immune transcriptomic analysis in bladder cancer reveals subtype specific immune gene expression patterns of prognostic relevance. *Oncotarget.* 2017;8(41):70982–1001. 10.18632/oncotarget.20237 29050337 PMC5642612

[70] Hurst CD Alder O Platt FM: Genomic Subtypes of Non-invasive Bladder Cancer with Distinct Metabolic Profile and Female Gender Bias in *KDM6A* Mutation Frequency. *Cancer Cell.* 2017;32(5):701–715.e7. 10.1016/j.ccell.2017.08.005 29136510 PMC5774674

[71] Hedegaard J Lamy P Nordentoft I: Comprehensive Transcriptional Analysis of Early-Stage Urothelial Carcinoma. *Cancer Cell.* 2016;30(1):27–42. 10.1016/j.ccell.2016.05.004 27321955

[72] Al Hussain TO Akhtar M: Molecular basis of urinary bladder cancer. *Adv Anat Pathol.* 2013;20(1):53–60. 10.1097/PAP.0b013e31827bd0ec 23232572

[73] Billerey C Chopin D Aubriot-Lorton MH: Frequent *FGFR3* mutations in papillary non-invasive bladder (pTa) tumors. *Am J Pathol.* 2001;158(6):1955–9. 10.1016/S0002-9440(10)64665-2 11395371 PMC1891972

[74] Liu X Zhang W Geng D: Clinical significance of fibroblast growth factor receptor-3 mutations in bladder cancer: a systematic review and meta-analysis. *Genet Mol Res.* 2014;13(1):1109–20. 10.4238/2014.February.20.12 24634132

[75] Pietzak EJ Bagrodia A Cha EK: Next-generation Sequencing of Nonmuscle Invasive Bladder Cancer Reveals Potential Biomarkers and Rational Therapeutic Targets. *Eur Urol.* 2017;72(6):952–9. 10.1016/j.eururo.2017.05.032 28583311 PMC6007852

[76] Allory Y Beukers W Sagrera A: Telomerase reverse transcriptase promoter mutations in bladder cancer: high frequency across stages, detection in urine, and lack of association with outcome. *Eur Urol.* 2014;65(2):360–6. 10.1016/j.eururo.2013.08.052 24018021

[77] Cheng L Davidson DD Wang M: Telomerase reverse transcriptase (TERT) promoter mutation analysis of benign, malignant and reactive urothelial lesions reveals a subpopulation of inverted papilloma with immortalizing genetic change. *Histopathology.* 2016;69(1):107–13. 10.1111/his.12920 26679899

[78] Wang CC Huang CY Jhuang YL: Biological significance of *TERT* promoter mutation in papillary urothelial neoplasm of low malignant potential. *Histopathology.* 2018;72(5):795–803. 10.1111/his.13441 29193225

[79] Kaasinen E Rintala E Hellström P: Factors explaining recurrence in patients undergoing chemoimmunotherapy regimens for frequently recurring superficial bladder carcinoma. *Eur Urol.* 2002;42(2):167–74. 10.1016/S0302-2838(02)00260-9 12160589

[80] Pode D Alon Y Horowitz AT: The mechanism of human bladder tumor implantation in an *in vitro* model. *J Urol.* 1986;136(2):482–6. 10.1016/S0022-5347(17)44926-3 3525861

[81] Barghi MR Rahmani MR Hosseini Moghaddam SM: Immediate intravesical instillation of mitomycin C after transurethral resection of bladder tumor in patients with low-risk superficial transitional cell carcinoma of bladder. *Urol J.* 2006;3(4):220–4. 10.22037/uj.v3i4.166 17559045

[82] Berrum-Svennung I Granfors T Jahnson S: A single instillation of epirubicin after transurethral resection of bladder tumors prevents only small recurrences. *J Urol.* 2008;179(1):101–5; discussion 105–6. 10.1016/j.juro.2007.08.166 17997459

[83] De Nunzio C Carbone A Albisinni S: Long-term experience with early single mitomycin C instillations in patients with low-risk non-muscle-invasive bladder cancer: prospective, single-centre randomised trial. *World J Urol.* 2011;29(4):517–21. 10.1007/s00345-011-0691-2 21594708

[84] Gudjónsson S Adell L Merdasa F: Should all patients with non-muscle-invasive bladder cancer receive early intravesical chemotherapy after transurethral resection? The results of a prospective randomised multicentre study. *Eur Urol.* 2009;55(4):773–80. 10.1016/j.eururo.2009.01.006 19153001

[85] Kang M Jeong CW Kwak C: Single, immediate postoperative instillation of chemotherapy in non-muscle invasive bladder cancer: a systematic review and network meta-analysis of randomized clinical trials using different drugs. *Oncotarget.* 2016;7(29):45479–88. 10.18632/oncotarget.9991 27323781 PMC5216735

[86] Perlis N Zlotta AR Beyene J: Immediate post-transurethral resection of bladder tumor intravesical chemotherapy prevents non-muscle-invasive bladder cancer recurrences: an updated meta-analysis on 2548 patients and quality-of-evidence review. *Eur Urol.* 2013;64(3):421–30. 10.1016/j.eururo.2013.06.009 23830475

[87] Solsona E Iborra I Ricós JV: Effectiveness of a single immediate mitomycin C instillation in patients with low risk superficial bladder cancer: short and long-term followup. *J Urol.* 1999;161(4):1120–3. 10.1016/S0022-5347(01)61606-9 10081851

[88] Sylvester RJ Oosterlinck W: An immediate instillation after transurethral resection of bladder tumor in non-muscle-invasive bladder cancer: has the evidence changed? *Eur Urol.* 2009;56(1):43–5. 10.1016/j.eururo.2009.03.074 19375216

[89] Sylvester RJ Oosterlinck W Holmang S: Systematic Review and Individual Patient Data Meta-analysis of Randomized Trials Comparing a Single Immediate Instillation of Chemotherapy After Transurethral Resection with Transurethral Resection Alone in Patients with Stage pTa-pT1 Urothelial Carcinoma of the Bladder: Which Patients Benefit from the Instillation? *Eur Urol.* 2016;69(2):231–44. 10.1016/j.eururo.2015.05.050 26091833

[90] Elmamoun MH Christmas TJ Woodhouse CR: Destruction of the bladder by single dose Mitomycin C for low-stage transitional cell carcinoma (TCC)--avoidance, recognition, management and consent. *BJU Int.* 2014;113(5b):E34–8. 10.1111/bju.12340 24053461

[91] Oddens JR van der Meijden AP Sylvester R: One immediate postoperative instillation of chemotherapy in low risk Ta, T1 bladder cancer patients. Is it always safe? *Eur Urol.* 2004;46(3):336–8. 10.1016/j.eururo.2004.05.003 15306104

[92] Bosschieter J Nieuwenhuijzen JA van Ginkel T: Value of an Immediate Intravesical Instillation of Mitomycin C in Patients with Non-muscle-invasive Bladder Cancer: A Prospective Multicentre Randomised Study in 2243 patients. *Eur Urol.* 2018;73(2):226–32. 10.1016/j.eururo.2017.06.038 28705539

[93] Böhle A Jocham D Bock PR: Intravesical bacillus Calmette-Guerin versus mitomycin C for superficial bladder cancer: a formal meta-analysis of comparative studies on recurrence and toxicity. *J Urol.* 2003;169(1):90–5. 10.1016/S0022-5347(05)64043-8 12478111

[94] Malmström PU Sylvester RJ Crawford DE: An individual patient data meta-analysis of the long-term outcome of randomised studies comparing intravesical mitomycin C versus bacillus Calmette-Guérin for non-muscle-invasive bladder cancer. *Eur Urol.* 2009;56(2):247–56. 10.1016/j.eururo.2009.04.038 19409692

[95] Shelley MD Mason MD Kynaston H: Intravesical therapy for superficial bladder cancer: a systematic review of randomised trials and meta-analyses. *Cancer Treat Rev.* 2010;36(3):195–205. 10.1016/j.ctrv.2009.12.005 20079574

[96] Shelley MD Wilt TJ Court J: Intravesical bacillus Calmette-Guérin is superior to mitomycin C in reducing tumour recurrence in high-risk superficial bladder cancer: a meta-analysis of randomized trials. *BJU Int.* 2004;93(4):485–90. 10.1111/j.1464-410X.2003.04655.x 15008714

[97] Chou R Selph S Buckley DI: Intravesical Therapy for the Treatment of Nonmuscle Invasive Bladder Cancer: A Systematic Review and Meta-Analysis. *J Urol.* 2017;197(5):1189–99. 10.1016/j.juro.2016.12.090 28027868

[98] Brausi M Oddens J Sylvester R: Side effects of Bacillus Calmette-Guérin (BCG) in the treatment of intermediate- and high-risk Ta, T1 papillary carcinoma of the bladder: results of the EORTC genito-urinary cancers group randomised phase 3 study comparing one-third dose with full dose and 1 year with 3 years of maintenance BCG. *Eur Urol.* 2014;65(1):69–76. 10.1016/j.eururo.2013.07.021 23910233

[99] Böhle A Bock PR: Intravesical bacille Calmette-Guérin versus mitomycin C in superficial bladder cancer: formal meta-analysis of comparative studies on tumor progression. *Urology.* 2004;63(4):682–6; discussion 686–7. 10.1016/j.urology.2003.11.049 15072879

[100] Kamat AM Sylvester RJ Böhle A: Definitions, End Points, and Clinical Trial Designs for Non-Muscle-Invasive Bladder Cancer: Recommendations From the International Bladder Cancer Group. *J Clin Oncol.* 2016;34(16):1935–44. 10.1200/JCO.2015.64.4070 26811532 PMC5321095

[101] Barlow LJ Benson MC: Experience with newer intravesical chemotherapy for high-risk non-muscle-invasive bladder cancer. *Curr Urol Rep.* 2013;14(2):65–70. 10.1007/s11934-013-0312-2 23378162

[102] Di Lorenzo G Perdonà S Damiano R: Gemcitabine versus bacille Calmette-Guérin after initial bacille Calmette-Guérin failure in non-muscle-invasive bladder cancer: a multicenter prospective randomized trial. *Cancer.* 2010;116(8):1893–900. 10.1002/cncr.24914 20162706

[103] McKiernan JM Holder DD Ghandour RA: Phase II trial of intravesical nanoparticle albumin bound paclitaxel for the treatment of nonmuscle invasive urothelial carcinoma of the bladder after bacillus Calmette-Guérin treatment failure. *J Urol.* 2014;192(6):1633–8. 10.1016/j.juro.2014.06.084 24996128

[104] Porten SP Leapman MS Greene KL: Intravesical chemotherapy in non-muscle-invasive bladder cancer. *Indian J Urol.* 2015;31(4):297–303. 10.4103/0970-1591.166446 26604440 PMC4626913

[105] Shelley MD Jones G Cleves A: Intravesical gemcitabine therapy for non-muscle invasive bladder cancer (NMIBC): a systematic review. *BJU Int.* 2012;109(4):496–505. 10.1111/j.1464-410X.2011.10880.x 22313502

[106] Steinberg G Bahnson R Brosman S: Efficacy and safety of valrubicin for the treatment of Bacillus Calmette-Guerin refractory carcinoma *in situ* of the bladder. The Valrubicin Study Group. *J Urol.* 2000;163(3):761–7. 10.1016/S0022-5347(05)67799-3 10687972

[107] Sternberg IA Dalbagni G Chen LY: Intravesical gemcitabine for high risk, nonmuscle invasive bladder cancer after bacillus Calmette-Guérin treatment failure. *J Urol.* 2013;190(5):1686–91. 10.1016/j.juro.2013.04.120 23665400

[108] Mathieu R Lucca I Rouprêt M: The prognostic role of lymphovascular invasion in urothelial carcinoma of the bladder. *Nat Rev Urol.* 2016;13(8):471–9. 10.1038/nrurol.2016.126 27431340

[109] Martin-Doyle W Leow JJ Orsola A: Improving selection criteria for early cystectomy in high-grade t1 bladder cancer: a meta-analysis of 15,215 patients. *J Clin Oncol.* 2015;33(6):643–50. 10.1200/JCO.2014.57.6967 25559810

[110] Willis DL Fernandez MI Dickstein RJ: Clinical outcomes of cT1 micropapillary bladder cancer. *J Urol.* 2015;193(4):1129–34. 10.1016/j.juro.2014.09.092 25254936 PMC4687395

[111] Segal R Yafi FA Brimo F: Prognostic factors and outcome in patients with T1 high-grade bladder cancer: can we identify patients for early cystectomy? *BJU Int.* 2012;109(7):1026–30. 10.1111/j.1464-410X.2011.10462.x 21883838

[112] Mally AD Tin AL Lee JK: Clinical Outcomes of Patients With T1 Nested Variant of Urothelial Carcinoma Compared to Pure Urothelial Carcinoma of the Bladder. *Clin Genitourin Cancer.* 2017; pii: S1558-7673(17)30199-4. 10.1016/j.clgc.2017.07.002 28802887 PMC5767538

[113] Ark JT Keegan KA Barocas DA: Incidence and predictors of understaging in patients with clinical T1 urothelial carcinoma undergoing radical cystectomy. *BJU Int.* 2014;113(6):894–9. 10.1111/bju.12245 24053444 PMC3874077

[114] Oughton JB Poad H Twiddy M: Radical cystectomy (bladder removal) against intravesical BCG immunotherapy for high-risk non-muscle invasive bladder cancer (BRAVO): a protocol for a randomised controlled feasibility study. *BMJ Open.* 2017;7(8):e017913. 28801444 10.1136/bmjopen-2017-017913 PMC5724134

[115] Mak RH Hunt D Shipley WU: Long-term outcomes in patients with muscle-invasive bladder cancer after selective bladder-preserving combined-modality therapy: a pooled analysis of Radiation Therapy Oncology Group protocols 8802, 8903, 9506, 9706, 9906, and 0233. *J Clin Oncol.* 2014;32(34):3801–9. 10.1200/JCO.2014.57.5548 25366678 PMC4239302

[116] Choudhury A Nelson LD Teo MT: MRE11 expression is predictive of cause-specific survival following radical radiotherapy for muscle-invasive bladder cancer. *Cancer Res.* 2010;70(18):7017–26. 10.1158/0008-5472.CAN-10-1202 20843819 PMC2941719

[117] Laurberg JR Brems-Eskildsen AS Nordentoft I: Expression of TIP60 (tat-interactive protein) and MRE11 (meiotic recombination 11 homolog) predict treatment-specific outcome of localised invasive bladder cancer. *BJU Int.* 2012;110(11 Pt C):E1228–36. 10.1111/j.1464-410X.2012.11564.x 23046361

[118] Grossman HB Natale RB Tangen CM: Neoadjuvant chemotherapy plus cystectomy compared with cystectomy alone for locally advanced bladder cancer. *N Engl J Med.* 2003;349(9):859–66. 10.1056/NEJMoa022148 12944571

[119] Advanced Bladder Cancer (ABC) Meta-analysis Collaboration: Neoadjuvant chemotherapy in invasive bladder cancer: update of a systematic review and meta-analysis of individual patient data advanced bladder cancer (ABC) meta-analysis collaboration. *Eur Urol.* 2005;48(2):202–5; discussion 205–6. 10.1016/j.eururo.2005.04.006 15939524

[120] Yuh BE Ruel N Wilson TG: Pooled analysis of clinical outcomes with neoadjuvant cisplatin and gemcitabine chemotherapy for muscle invasive bladder cancer. *J Urol.* 2013;189(5):1682–6. 10.1016/j.juro.2012.10.120 23123547 PMC3926865

[121] International Collaboration of Trialists, Medical Research Council Advanced Bladder Cancer Working Party (now the National Cancer Research Institute Bladder Cancer Clinical Studies Group), European Organisation for Research and Treatment of Cancer Genito-Urinary Tract Cancer Group, *et al. *: International phase III trial assessing neoadjuvant cisplatin, methotrexate, and vinblastine chemotherapy for muscle-invasive bladder cancer: long-term results of the BA06 30894 trial. *J Clin Oncol.* 2011;29(16):2171–7. 10.1200/JCO.2010.32.3139 21502557 PMC3107740

[122] Culp SH Dickstein RJ Grossman HB: Refining patient selection for neoadjuvant chemotherapy before radical cystectomy. *J Urol.* 2014;191(1):40–7. 10.1016/j.juro.2013.07.061 23911605 PMC4158919

[123] Moschini M Soria F Klatte T: Validation of Preoperative Risk Grouping of the Selection of Patients Most Likely to Benefit From Neoadjuvant Chemotherapy Before Radical Cystectomy. *Clin Genitourin Cancer.* 2017;15(2):e267–e273. 10.1016/j.clgc.2016.07.014 27530435

[124] Sternberg CN Skoneczna I Kerst JM: Immediate versus deferred chemotherapy after radical cystectomy in patients with pT3-pT4 or N+ M0 urothelial carcinoma of the bladder (EORTC 30994): an intergroup, open-label, randomised phase 3 trial. *Lancet Oncol.* 2015;16(1):76–86. 10.1016/S1470-2045(14)71160-X 25498218

[125] Dong F Shen Y Gao F: Nomograms to Predict Individual Prognosis of Patients with Primary Small Cell Carcinoma of the Bladder. *J Cancer.* 2018;9(7):1152–64. 10.7150/jca.23344 29675096 PMC5907663

[126] Kouba EJ Cheng L: Understanding the Genetic Landscape of Small Cell Carcinoma of the Urinary Bladder and Implications for Diagnosis, Prognosis, and Treatment: A Review. *JAMA Oncol.* 2017;3(11):1570–8. 10.1001/jamaoncol.2016.7013 28334324

[127] Millis SZ Bryant D Basu G: Molecular profiling of infiltrating urothelial carcinoma of bladder and nonbladder origin. *Clin Genitourin Cancer.* 2015;13(1):e37–49. 10.1016/j.clgc.2014.07.010 25178641

[128] Zheng X Liu D Fallon JT: Distinct genetic alterations in small cell carcinoma from different anatomic sites. *Exp Hematol Oncol.* 2015;4:2. 10.1186/2162-3619-4-2 25937998 PMC4417281

[129] Siefker-Radtke AO Kamat AM Grossman HB: Phase II clinical trial of neoadjuvant alternating doublet chemotherapy with ifosfamide/doxorubicin and etoposide/cisplatin in small-cell urothelial cancer. *J Clin Oncol.* 2009;27(16):2592–7. 10.1200/JCO.2008.19.0256 19414678 PMC4879720

